# Supplementary material for: Associations of cerebral oxygenation with hemoglobin levels evaluated by near-infrared spectroscopy in hemodialysis patients
Source: PLoS One. 2020 Aug 10;15(8):e0236720. doi: 10.1371/journal.pone.0236720 (PMC7416957; doi:10.1371/journal.pone.0236720)
Supplement: Attachment — Submitted filename: Response to Journal Requirement 2.docx [file pone.0236720.s001.docx]

Response to the journal requirements

We appreciate your careful review of our manuscript and thank you for the opportunity to submit our revised manuscript and the point-by-point responses to the journal requirements. We hope that we have satisfactorily addressed all issues raised by the journal requirements.

Comment

1. We note that your author list was updated during the revision process. Please complete our Authorship Change Form by following this link: <http://journals.plos.org/plosone/s/file?id=13d0/plos-one-change-to-authorship-form.docx>. You may return the form as an attachment by emailing [plosone@plos.org](mailto:plosone@plos.org) or by uploading it as a submission file labeled with the file type ‘Other’. Please note that if your manuscript is accepted, we will not be able to complete the publication process without the completed form.

Response

As suggested by Journal Requirement, we completed our Authorship Change Form and uploaded it as a submission file in the revised manuscript.

Comment

2. Thank you for explaining that Hakuyukai Medical Corporation didn't provide financial support for your study. We note that several authors are affiliated to clinics run by Hakuyukai Medical Corporation, however, so for full transparency we would like this to be noted in your Funding and Competing Interests statements.

Can you please confirm whether the following proposed Funding statement is accurate and suitable to appear alongside your manuscript?

"This work was supported by grants from the Japanese Association of Dialysis Physicians (JADP Grant 2017-9), The Kidney Foundation, Japan (JKFB 18-7), and JSPS KAKENHI under Grant No. JP20K11534 to SO. Additionally, Hakuyukai Medical Corporation provided support in the form of salaries for authors KH, TA, SI, HS, and AW. The specific roles of these authors are articulated in the ‘author contributions’ section. The funders had no role in study design, data collection and analysis, decision to publish, or manuscript preparation."

Response

We confirmed the proposed Funding statement is accurate and suitable.

Comment

3. And, can you please confirm whether the following proposed Competing Interests statement is accurate and suitable to appear alongside your manuscript?

"Hakuyukai Medical Corporation provided support in the form of salaries for authors KH, TA, SI, HS, and AW. There are no patents, products in development or marketed products associated with this research to declare. This does not alter our adherence to PLOS ONE policies on sharing data and materials."

Response

We confirmed the proposed Funding statement is accurate and suitable.

We hope that our revised manuscript is now suitable for publication in your highly esteemed journal.
